# Endovascular preoperative embolization of carotid paraganglioma: A case report

**DOI:** 10.1016/j.radcr.2025.09.088

**Published:** 2025-10-31

**Authors:** Hussein-Choukri Ahmanna, Badr Boutakioute, Ayoub El Hajjami, Yazen El Badri, Youssef Bouktib, Mariem Idrissi Ouali, Najat Cherif Idrissi El Ganouni

**Affiliations:** Department of Radiology, Mohammed VI University Medical Center, Marrakech, Morocco

**Keywords:** Carotid body tumor, Paraganglioma, Embolization, Endovascular, Preoperative

## Abstract

Carotid body paragangliomas are highly vascular tumors of the head and neck. Surgical resection is the gold standard treatment, and preoperative embolization has been proposed to reduce intraoperative bleeding and facilitate tumor removal. We present the case of a 33-year-old woman with a rapidly enlarging left cervical mass. CT and MRI demonstrated a lesion at the carotid bifurcation with splaying of the internal and external carotid arteries ('lyre sign'), consistent with a Shamblin group III carotid body tumor. Digital subtraction angiography revealed intense tumoral blush supplied mainly by branches of the external carotid artery. Selective embolization with 400-µm Embosphere® Microspheres (Merit Medical Systems, South Jordan, UT, USA) was performed 24 hours before surgery, achieving approximately 50% devascularization without complications. Complete surgical excision was subsequently achieved with minimal intraoperative bleeding and no need for transfusion. Postoperative recovery was uneventful. Preoperative endovascular embolization is a safe and effective adjunct in the management of carotid body paragangliomas. It reduces vascularity, facilitates surgical dissection, and may decrease perioperative morbidity.

## Introduction

Paragangliomas of the head and neck are rare tumors arising from paraganglionic tissue derived from neural crest cells. The most common locations are the carotid body, jugular foramen, and vagus nerve [1]. Surgical resection remains the primary treatment, aiming for complete excision. Given the rich vascularity of these tumors, preoperative embolization has been proposed as an adjuvant to reduce intraoperative bleeding and facilitate surgery [2]. We report a case of carotid body paraganglioma successfully managed with preoperative endovascular embolization followed by surgical resection.

## Objective

The objective of this report is to illustrate the imaging findings, embolization technique, and the role of preoperative embolization in the successful surgical management of carotid body paraganglioma.

## Case report

**Fig. 1:**
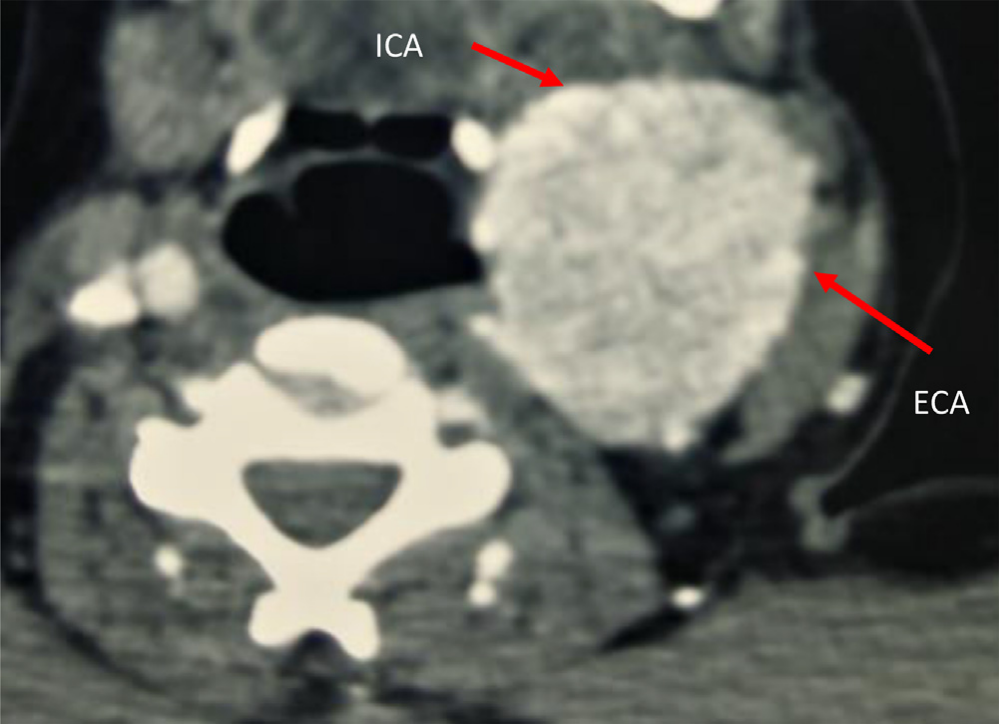
Axial CT scan (arterial phase) showing a large left cervical mass at the carotid bifurcation with intense enhancement. The internal carotid artery (ICA) and external carotid artery (ECA) are splayed apart (lyre sign).

A 33-year-old woman with no significant past medical history presented with a rapidly growing, firm left cervical mass. There were no overlying skin changes or palpable lymphadenopathy. CT scan revealed a mass at the carotid bifurcation with splaying of the internal and external carotid arteries (lyre sign), showing avid arterial enhancement and >270° circumferential contact with the internal carotid artery, consistent with a Shamblin group III tumor (Fig. 1).

**Fig. 2:**
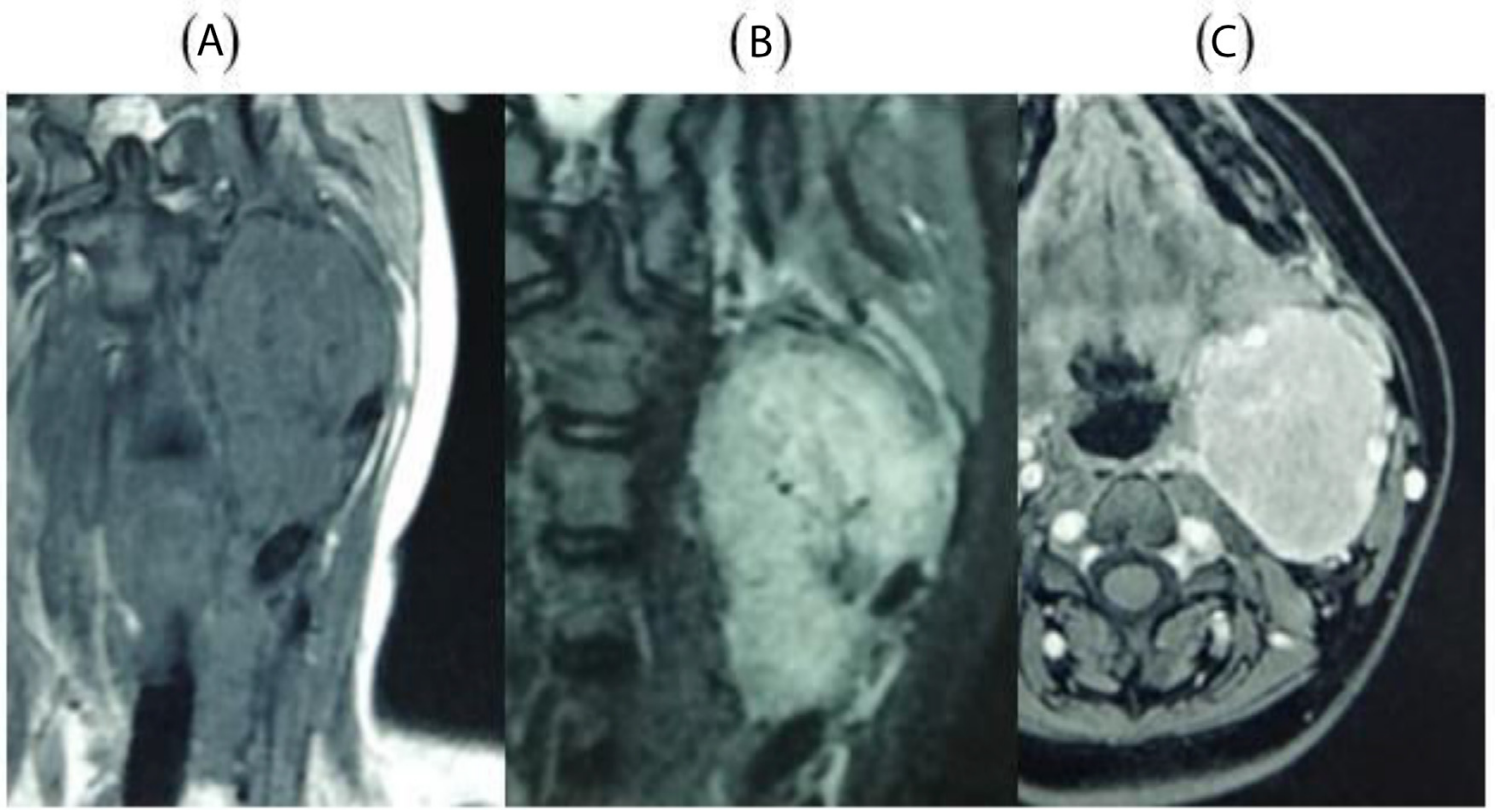
MRI: (A) Coronal T1-weighted: isointense mass; (B) Coronal T2-weighted: hyperintense mass; (C) Axial T1 post-gadolinium: avid enhancement. ICA and ECA are labeled.

MRI demonstrated an isointense mass on T1-weighted images, hyperintense on T2, with avid enhancement following gadolinium administration (Fig. 2).

**Fig. 3:**
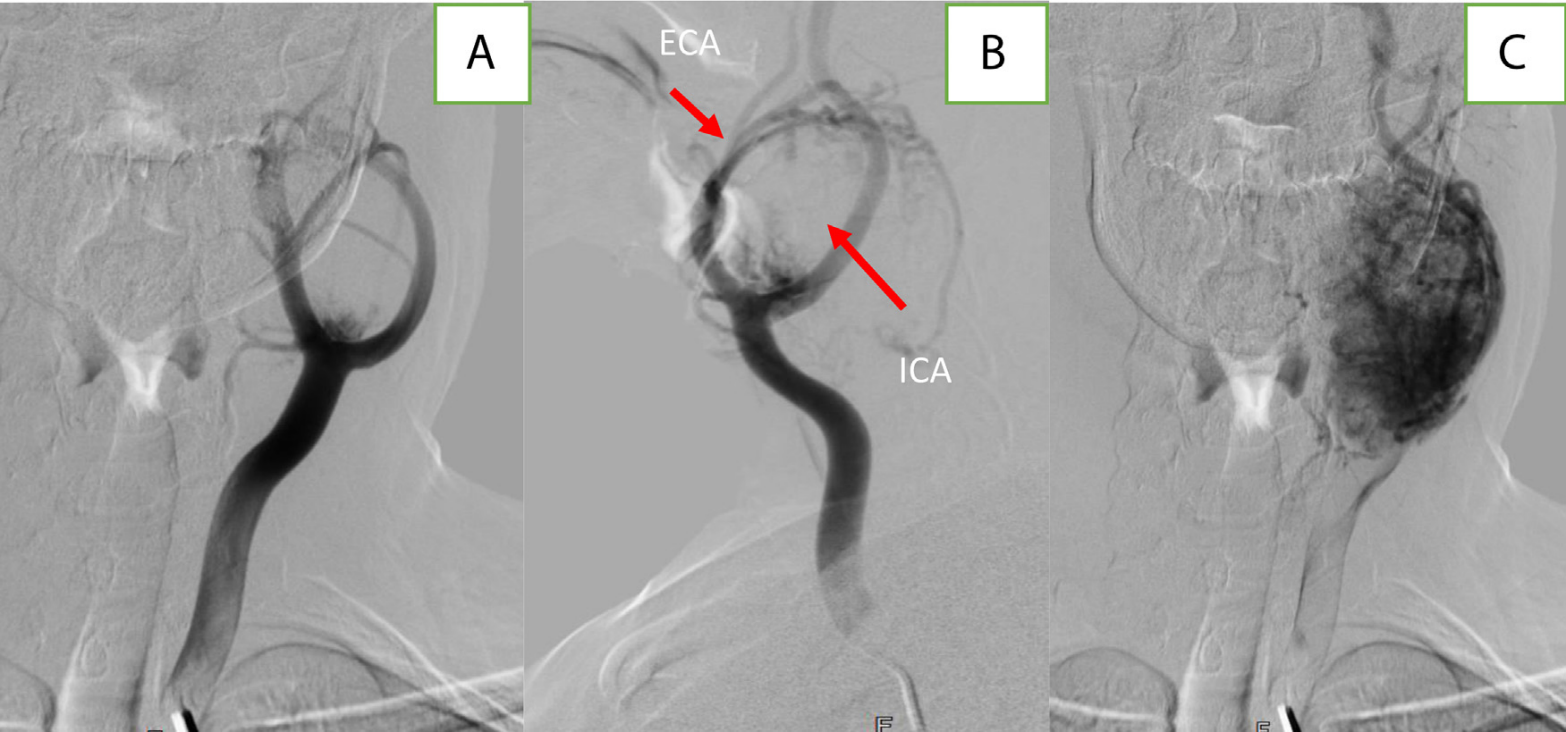
DSA demonstrating “the lyre sign” on frontal (A) and (B) oblique view with an important tumoral blush with 2 main branches that feed the tumor blush (C).

Digital subtraction angiography using a 5F diagnostic catheter demonstrated intense tumor blush, mainly supplied by branches of the external carotid artery, notably the ascending pharyngeal and lingual arteries (Fig. 3).

**Fig. 4:**
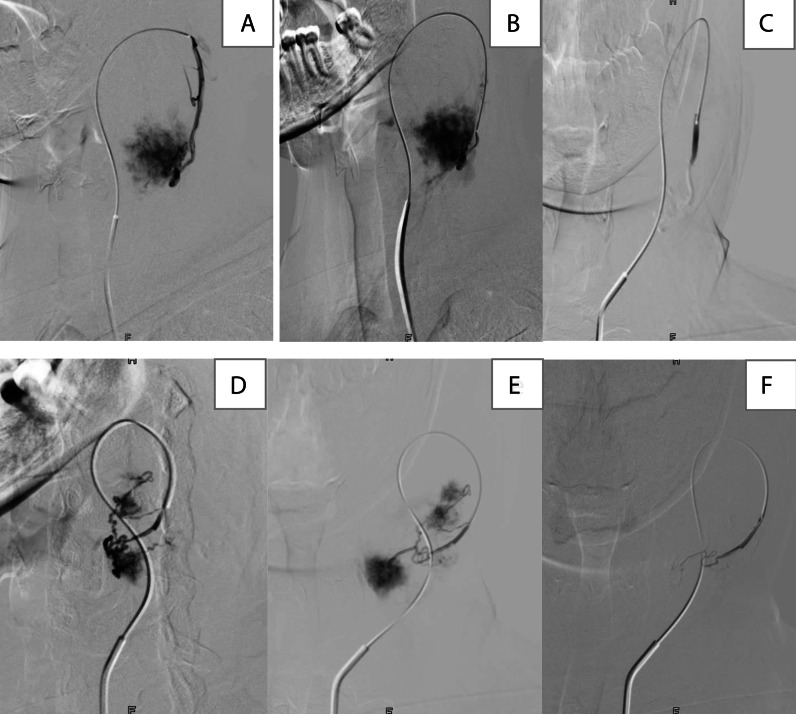
Selective arteriograms pre embolization (A and B) of the first branch of the external carotid artery responsible for the vascularization of the paraganglioma, (C) post embolization angiogram showing complete devascularization Pre embolization supra selective arteriograms (C and D) of the second branch showing a residual blush in the lower aspect of the mass, (E) Post embolization angiogram control showing a significant reduction of tumoral blush.

An embolization was performed 24 hours before the surgery, a microcatheter is placed through the diagnostic catheter and advanced into selective branches responsible for tumoral blush, where selective arteriograms were performed for improved tumor visualization, Particles of embosphers of 400 microns were mixed with contrast then attached to the microcatheter and injected in pulses until there were a significant cessation of flow and reflux of contrast along the microcatheter, a final angiogram was performed evaluating the degree of embolization that was around 50% with no further complications (Fig. 4).

**Fig. 5:**
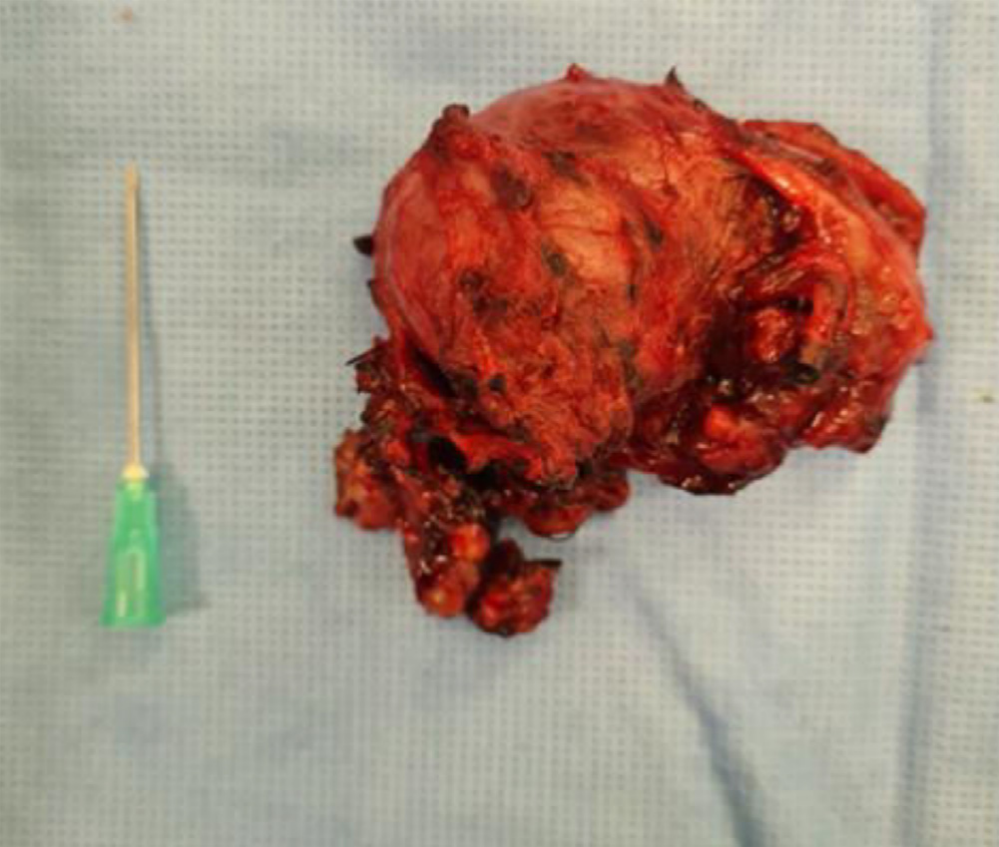
Macroscopic specimen of the carotid body paraganglioma after surgical excision, achieved with minimal blood loss and no transfusion requirement.

Twenty-four hours later, complete surgical resection was performed with minimal intraoperative bleeding, no need for transfusion, and uneventful recovery (Fig. 5).

## Discussion

Carotid body tumors (CBTs) represent 60–78% of head and neck paragangliomas, with a reported malignancy rate of 6–10% [3]. Imaging with CT and MRI plays a crucial role in diagnosis. On CT, CBTs appear as well-defined, hypervascular masses at the carotid bifurcation, causing splaying of the internal and external carotid arteries. Large lesions may extend to the suprahyoid neck and displace the internal carotid artery posteriorly, helping distinguish them from vagal paragangliomas [4,5]. On MRI, the characteristic “salt-and-pepper” appearance is observed due to intratumoral flow voids. Angiography is primarily performed for embolization planning, demonstrating intense tumor blush and multiple feeding arteries [6].

Embolic materials include polyvinyl alcohol, Embosphere® microspheres, n-butyl cyanoacrylate, and ethyl vinyl alcohol copolymer (Onyx). The timing of embolization is usually 24-72 hours before surgery to allow thrombosis and necrosis while avoiding recanalization and collateral vessel formation [7].

Several studies have evaluated the role of preoperative embolization in carotid body tumor (CBT) surgery. A systematic review and meta-analysis (Jackson et al. [8]) reported a mean reduction of intraoperative blood loss ranging from 300 to 500 mL and a decrease in operative time of approximately 30-60 minutes in embolized patients compared with those who underwent surgery alone. In addition, the transfusion rate was significantly lower in the embolization group. Conversely, a more recent Turkish series (Deniz et al., 2020) [9] found no statistically significant differences in blood loss, operative duration, or morbidity, highlighting the ongoing controversy. Complications of embolization are rare but have been described, including transient cranial nerve palsies, inadvertent embolization of the internal carotid artery, and stroke, with an overall complication rate below 5% in most large series.

The review by Romagnoli et al. [10] further emphasized that the utility of preoperative embolization remains debated, with technical success rates consistently high (>95%), but clinical benefits varying across institutions.

In our case, despite achieving only about 50% devascularization, surgical excision of a large Shamblin III CBT was completed without major bleeding or transfusion, which makes this case remarkable. It illustrates that even partial embolization can provide significant surgical advantages in selected cases.

## Conclusion

Carotid body paragangliomas are rare but highly vascular tumors arising in close proximity to major vessels. Diagnosis relies on CT and MRI, with angiography essential for treatment planning. Preoperative embolization is a safe and useful adjunct to surgery, facilitating tumor excision by reducing vascularity and intraoperative morbidity.

## Patient consent

Written informed consent was obtained from the patient for the publication of this case report. The patient understand that their identity will remain confidential and that all efforts will be made to ensure anonymity.
